# Design and analysis of a new type of mobile ice cooling equipment for deep mine

**DOI:** 10.1038/s41598-023-47902-2

**Published:** 2023-11-21

**Authors:** Xingdong Zhao, Siyu Zhao, Ang Li

**Affiliations:** School of Resources and Civil Engineering, Northeastern University, Shenyang, 110819 People’s Republic of China

**Keywords:** Energy science and technology, Engineering

## Abstract

In the background environment of the serious problem of high temperature heat damage in deep mining, some mines have complex and interlocking forms of roadway arrangement, with the innovative concept of cooling on demand as the principle, this paper develops a mobile ice cooling equipment, and introduces and explains the equipment from the perspective of principle, composition and dimensions. and uses Comsol simulation software to simulate and analyze the main heat exchange process of the mobile ice cooling equipment under the conditions of two cooling sources, obtains quantitative results on the finned tube arrangement parameters and the heat exchange cooling effect of the equipment under ideal conditions, which provides data for the optimization and upgrading of this mobile ice cooling equipment. The results show that the mobile cooling equipment is capable of feeding the desired temperature of the cooling air into deep mine, and with flexible, convenient, efficient, and cost effective. This research and development is a new exploration of deep ventilation and cooling technology and equipment means, puts forward a new concept, accumulates valuable experience, and lays the foundation for the subsequent related research and optimization.

## Introduction

As the depth of mining continues to increase worldwide, the problem of high temperature heat damage from deep mining is becoming more and more serious as a result^1^. In South Africa, the mining face of the President's Gold Mine in South Africa is over 3000 m deep and the virgin rock temperature is as high as 63 °C. The Robinson Gold Mine is mined at a depth of 2700 m and the virgin rock temperature is 41 °C. The Mount Isa Copper Mine in northern Australia has a virgin rock temperature of 60 °C at its depth of 2000 m. The Petrov Mine in the former Soviet Union is mined at a depth of 1200 m and the virgin rock temperature reaches 52 °C. The Ibbenbüren Mine in Germany is mined at a depth of 1530 m, the rock temperature at the bottom of the shaft was as high as 60 °C. At a depth of 2400 m at the Creighton mine in Canada, the original rock temperature was 48 °C^2^. Suncun coal mine of China at a depth of 1050 m, the rock temperature reaches 40–45 °C, and the wind temperature at the mining face reaches 32–34 °C. The temperature of the underground part of the mine has reached 40 °C in Hongtoushan copper mine and Dongguashan copper mine. The mine at a depth of 800 m has reached 34.5 °C in Sanshandao gold mine, and the temperature of the mine at a depth of 1800 m will exceed 60 °C.

With the increase in mining depth, the deep rock temperature rises significantly, resulting in higher air temperatures at the mining face and seriously deteriorating the underground operating environment^3^. Therefore, the world's high temperature mining countries began to study mine cooling technology in the last century, which refrigeration technology is the most important means of cooling in deep mining, more than 15 countries have used large refrigeration systems in deep mining areas^4,5^.

The world's first mine air conditioning system was established in 1915 at the Morao Jorge gold mine in Brazil, with a centralised refrigeration station on the ground surface. 2000 m deep, with a surrounding rocks temperature of 50 °C, a piston chiller was used to cool the temperature from 30 to 6 °C^6^. In 1923, Pendleton Colliery in the UK was the first to install a chiller in the mining area to cool the air flow at the mining face. Germany was the first to install a chiller in the Radlod coal mine in 1924, with a mining depth of 968 m and a surrounding rocks temperature of 44 °C. The chiller was installed on the ground surface and could reduce the wind temperature from 22.5 to 19.5 °C. The copper mine at Isa Enterprises in Australia uses a refrigeration system to pre-cool the air entering the main air shaft, which can cool the air temperature from 26 to 14.6 °C. A refrigeration system was installed at the Cade mine in Canada to cool the 35 °C air temperature at a depth of 3000 m. South Africa began using centralised air conditioning in large mines in the 1960s, using ice cooling technology to cool the deepest Mponeng gold mine, the mine buried at a depth of 3.5 km at a temperature of around 60 °C. Through ventilation and frozen filler technology, the air temperature can be cooled to around 32 °C. The Soviet Union, Japan and other countries began to apply refrigeration cooling in the 1970s. China started to adopt refrigeration cooling technology in 1964. In 1979, the Fushun Branch and Wuhan Freezer Factory cooperated to develop the JKT-70 mining chiller unit for cooling the mining face. In 1994, the Xinwen Suncun Mine established China's first centralized ground cooling and air-conditioning system.

Around the world, mine air conditioning and cooling is growing in scale and technology is developing rapidly. However, in the actual production operations of many mining companies, cooling of the entire mine is not possible due to cost and technical constraints. The temperature in the deeper parts of the mine is so high that the cooling effect and cost of using the common spray or compression cooling equipment is no longer better suited to the actual requirements of the underground mining ^7^.

In addition, ventilation cooling in deep non-essential areas causes unnecessary consumption of energy, and many mines optimize and upgrade their ventilation cooling systems to ventilation on-demand with the intention of reducing energy consumption^8^. But it is equally important to have cooling on-demand for deep mining cooling, where only essential areas are ventilated and cooled locally according to the temperature requirements of the mining face in order to achieve a reduction in energy consumption^9^.

Therefore, it has practical importance and good prospect to consider the development of a new mobile ice cooling equipment for underground, which was the original intention and purpose of developing the Mobile Ice Cooling Combination Vehicle—“MICV”. Based on the new principle of cooling on-demand**.** The Mobile Ice Cooling Combined Vehicle can be used for localized ventilation and cooling in the required area of the shaft, and it is flexible, convenient, efficient, cost effective and has a controllable cooling source^10^, and achieve a relative balance between ventilation and cooling and rational use of energy in the shaft to the greatest extent possible.

## Equipment development

### Principle of equipment development

Mobile ice cooling equipment with ice and water mixed cold source as the medium, finned tube type heat exchanger as the core, variable frequency water pump as the control, one end connected to the extracted local fan, using the high melting heat of the ice media to cool the wind flow through the heat exchanger, to be able to send cooling air to the mining face, in order to ensure the normal thermal environment of the mining^11^.

### The basic structure of the equipment

**Figure 1:**
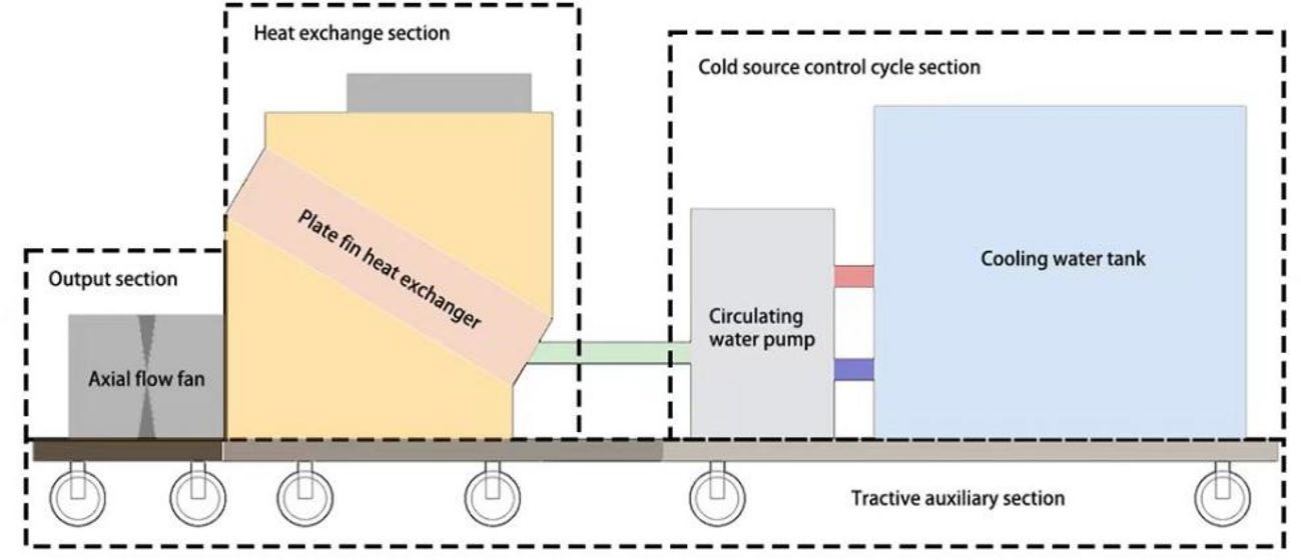
Schematic diagram of the components of a mobile ice media cooling plant.

Mobile ice cooling equipment is mainly divided into four unit structures: heat exchange part, cold source control cycle part, output part, traction auxiliary part, etc. The schematic is shown in Fig. 1.

**Figure 2:**
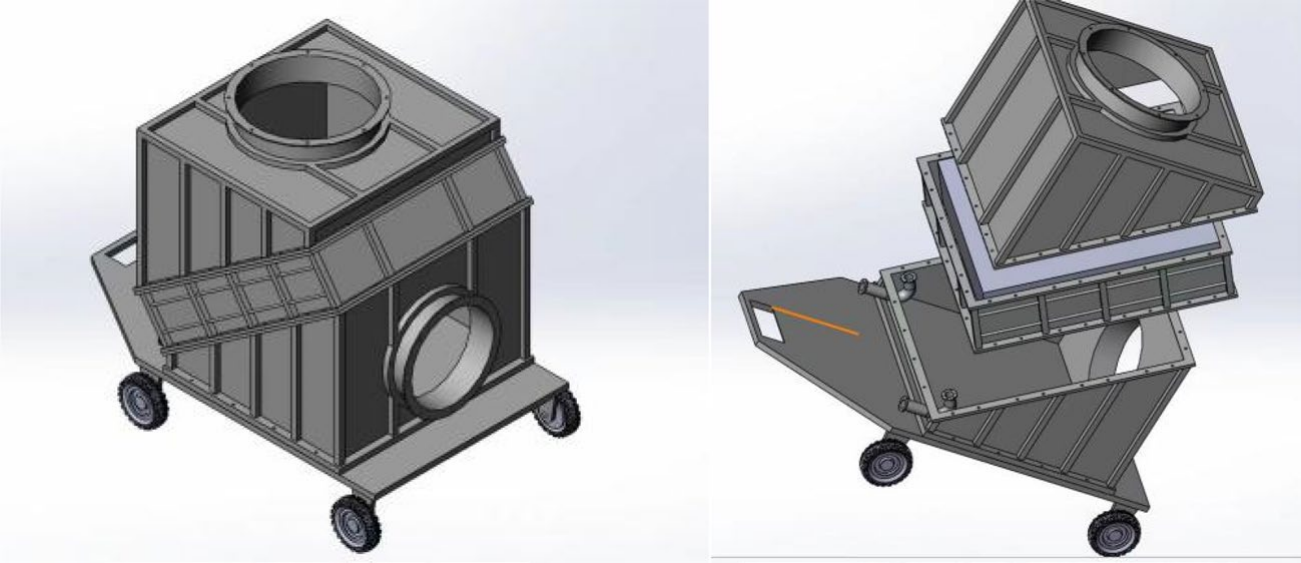
3D model of the main body of the mobile ice media cooling equipment.

The heat exchange part consists of the equipment enclosure and the finned tube heat exchanger, which is the most important component of the mobile ice media cooling equipment, mainly for heat transfer between the high temperature air flow and the cold source, the 3D model is shown in Fig. 2.

The cold source control circulation section consists of a variable frequency water pump, an ice tank and water pipes. The ice tank is filled with an ice and water mixture and ice is added at regular intervals to ensure the temperature of the cold source. The variable frequency water pump can provide a stable circulating cold source to the heat exchanger tube by controlling the flow rate of the cold source.

The output section is directly connected to the extraction ventilator, which directs the cooled cold air flow into the interior of the high temperature tunnel. The interface can also be connected with a suitable flexible duct depending on the downhole site conditions.

The traction aid consists of a base, wheels, tractor, fan support etc. which allows the equipment to travel freely and be easily assembled underground.

### Equipment design parameters dimensions

**Figure 3:**
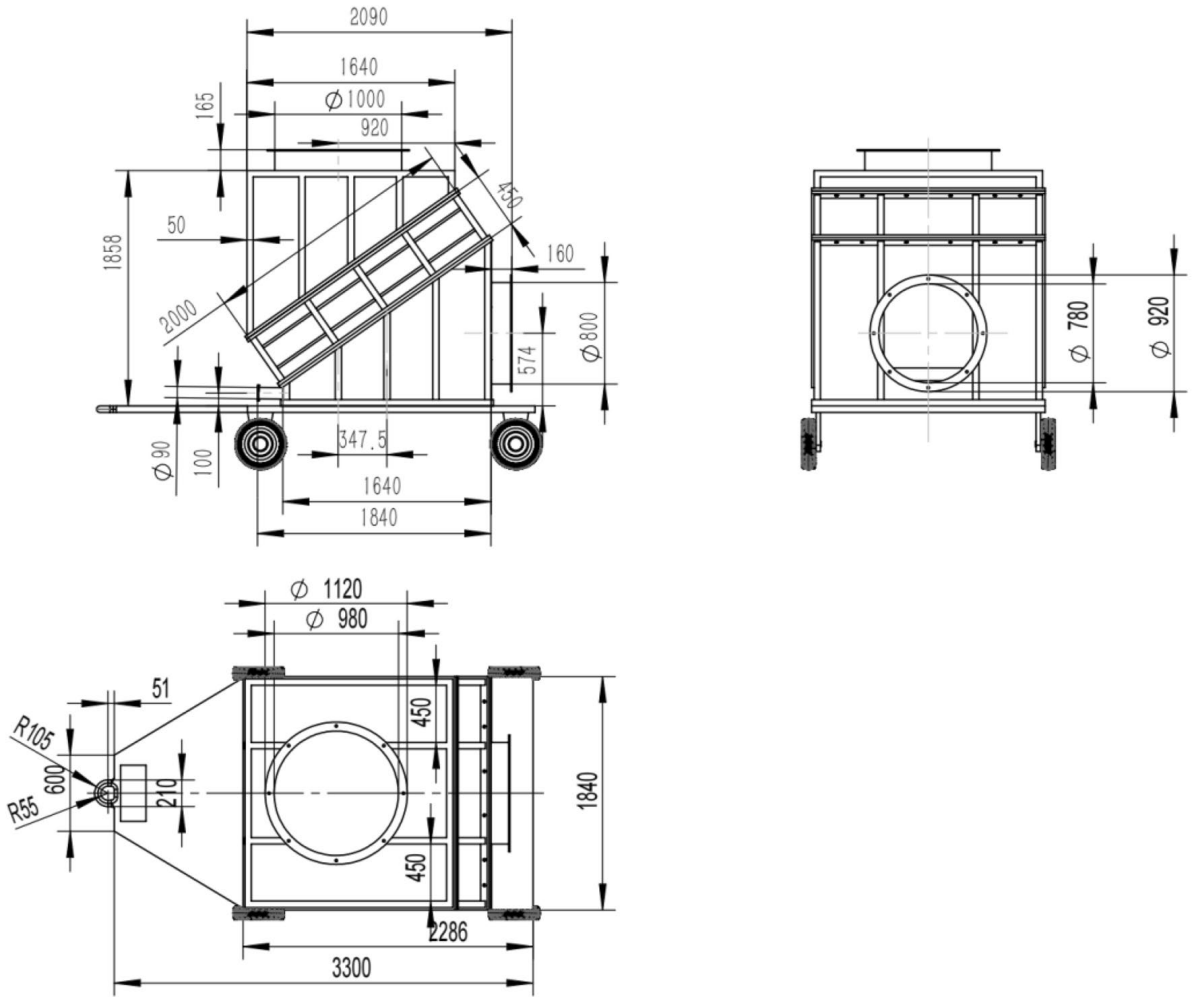
Three views of the mobile ice media cooling equipment.

The main design dimensions of the mobile ice media cooling equipment are shown in Fig. 3.

### Specific working process

The hot air flow from the underground mining face passes through the finned tube heat exchanger in the equipment, using the cold source in the tube to exchange heat for the hot air flow, thus realizing the purpose of cooling the high temperature air flow. Then the cold air is extracted by the local fan and discharged to the high temperature area of the mine roadway.

The cold water in the finned tube and the ice water in the tank complete the cycle through the pressure of the inverter pump, using the high melting heat of the ice to absorb heat from the circulating water and achieve cooling of the circulating water, thus obtaining a stable circulating cold source.

The above two circulation systems use the core heat exchange part as the hub to complete the multi-phase heat transfer between solid (ice)—liquid (water)—gas (wind flow).

## Computational simulation of mobile ice media cooling equipment

### Equipment core

**Figure 4:**
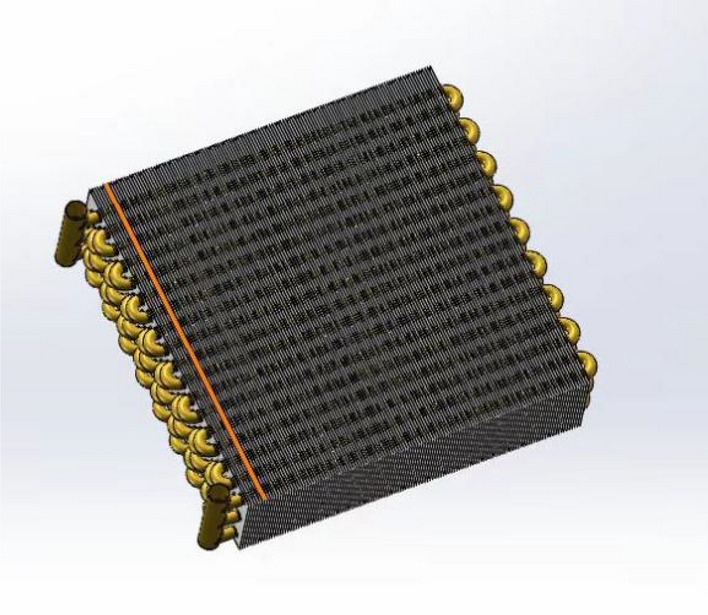
3D model of finned tube heat exchanger.

The core heat transfer part of the mobile ice cooling equipment is designed with finned tube heat exchangers, which are more effective than tube and finned tube heat exchangers for heat transfer between the high temperature air flow and the cold source. Firstly, the finned tube heat exchanger uses a compact fin arrangement to increase the heat transfer area in the limited space of the equipment. Secondly, the fins guide the air flow to enhance the convection effect between the fluids and enhance the heat transfer performance. The 3D model of the finned tube heat exchanger is shown in Fig. 4.

### Model establishment and boundary condition setting

In order to study the finned tube arrangement parameters and the heat exchange cooling effect of the equipment, this study uses Comsol Multiphysics simulation software to establish a local inter-fin air field model to simulate the temperature drop and the range of action when the air flow passes through the inter-fin air field^12^.

Comsol Multiphysics is a multi-physics field coupled simulation software based on advanced numerical methods, which can be used to study the parameter determination and optimization of finned tube heat exchangers, and can simulate the heat transfer effect of different temperature cooling sources, so as to finally determine reasonable equipment parameters.

**Figure 5:**
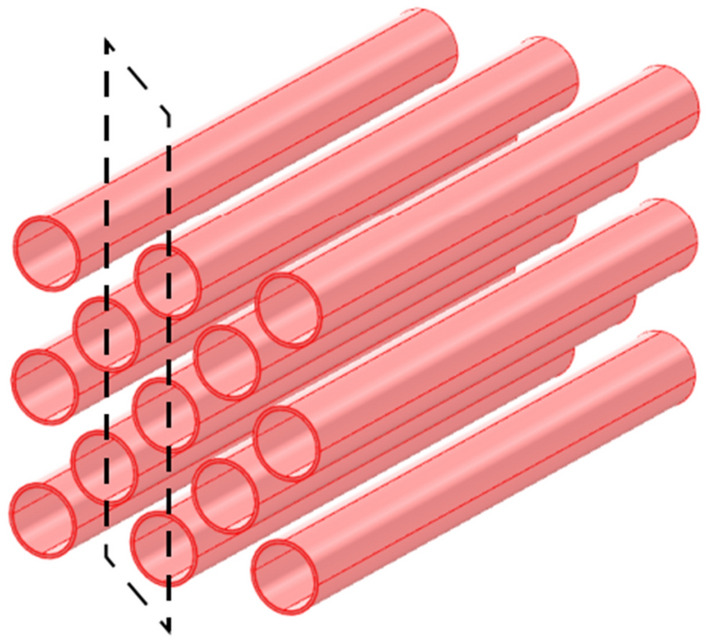
Schematic diagram for the selection of analogue units.

The selected area for the local inter-fin air field simulation unit established in this paper is shown in Fig. 5.

**Figure 6:**
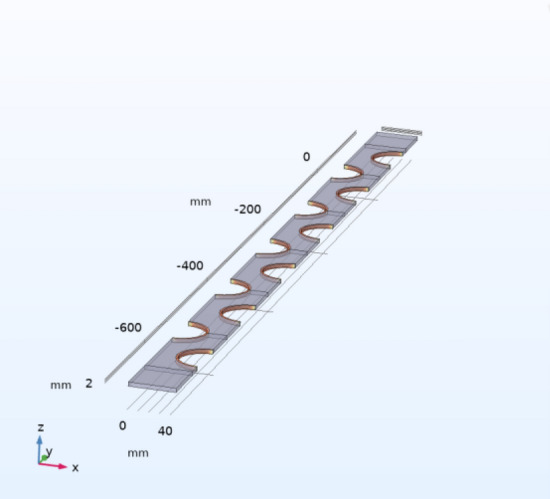
Simulation model diagram.

The model dimensional parameters are designed as follows: effective length of the heat exchanger tube L = 1600 mm. wall thickness of the heat exchanger tube δ_*1*_ = 2 mm. diameter of the heat exchanger tube d_*1*_ = 30 mm. fin spacing F_*p*_ = 2 mm. fin thickness δ_*F*_ = 2 mm. In order to take into account the heat transfer efficiency and corrosion resistance, the material of the finned tube heat exchanger is chosen as copper alloy. The model is shown in Fig. 6.

The turbulent k-ε model was used for the fluid flow model in this simulation, and a steady-state study model of non-isothermal incompressible flow was selected for the fluid heat transfer physical field. At the same time, it is also necessary to establish the mathematical model of the finned tube heat exchanger, which mainly applies the three major differential equations in fluid mechanics, namely, the mass conservation differential equation, the momentum conservation differential equation and the energy conservation differential equation.

It is worth noting that in deep high-temperature roadways, the air flow through the finned tube heat exchanger is usually in a turbulent state. In engineering applications and numerical simulation, the standard k − ε bipartite equation model is the most commonly used model with satisfactory results. Therefore, we use the standard k − ε two-way equation model to simulate the heat transfer results of finned tube heat exchangers.

However, although the k − ε model is suitable for fully developed turbulence models at high Reynolds numbers, it has high simulation accuracy. However, the fluid flow near the fin in the model is insufficient, resulting in a low Reynolds number and a flow state similar to laminar flow. If only k − ε model is used, the simulation error will be large. So we decided to use the wall function method to effectively supplement the k-ε model. The k − ε model can be modified and supplemented by using wall function in Comsol Multiphysics software. The specific equation is expressed as follows:

1. Continuity equation:

1 $$\frac{{\partial u_{i} }}{{\partial x_{i} }} = 0,i = 1,2,3$$

2. Momentum conservation equation:

2 $$\frac{{Du_{i} }}{Dt} = f_{i} - \frac{\partial p}{{\partial x_{i} }} + \frac{\partial }{{\partial x_{j} }}\left( {\frac{{\partial u_{i} }}{{\partial x_{j} }} - \overline{{u_{i} }} \overline{{u_{j} }} } \right)$$

3. Standard k equation:

3 $$\rho \frac{{\partial \left( {ku_{i} } \right)}}{{\partial x_{i} }} = \frac{\partial }{{\partial x_{j} }}\left[ {\left( {\mu + \frac{{\mu_{t} }}{{\sigma_{k} }}} \right)\frac{\partial k}{{\partial x_{j} }}} \right] + \tau_{ij} S_{ij} - \rho {\upvarepsilon } + \phi_{k}$$

4. Standard ε equation:

4 $$\rho \frac{{\partial \left( {{\upvarepsilon }u_{i} } \right)}}{{\partial x_{i} }} = \frac{\partial }{{\partial x_{j} }}\left[ {\left( {\mu + \frac{{\mu_{t} }}{{\sigma_{{\upvarepsilon }} }}} \right)\frac{{\partial {\upvarepsilon }}}{{\partial x_{j} }}} \right] + c_{{1{\upvarepsilon }}} \frac{{\upvarepsilon }}{k}\tau_{ij} s_{ij} - c_{{2{\upvarepsilon }}} f_{2} \rho \frac{{{\upvarepsilon }^{2} }}{k} + \phi_{k}$$

Among: k—Turbulent kinetic energy, m^2^/s^2^. ε—Dissipation rate of turbulent kinetic energy, m^2^/s^3^.${u}_{i}$—Velocity component, m/s. ${\upmu }_{t}$—Coefficient of viscosity,${\upmu }_{t}=\frac{{C}_{\mu }\uprho {k}^{2}}{\upvarepsilon }$. f_2_—Near-wall attenuation function,${\sigma }_{k}$, ${\sigma }_{\upvarepsilon }$—The Prandtl number of k, ε; ${c}_{u}$,${c}_{1\upvarepsilon }$, ${c}_{2\upvarepsilon }$—constant, respectively equal to 0.09, 1.44, 1.92.

In this paper, according to the actual temperature range data of the underground mine cold source, we choose 4 °C and 10 °C cold source temperature as the critical temperature for comparative study, grid the calculation units with symmetry and periodicity, and set the fluid area of the inlet and outlet section to prevent the influence of the inlet and outlet fluid. In order to solve the calculation, we also make some conditional assumptions and numerical Settings:

1. Suppose the air flow is an incompressible fluid
2. In-flow air velocity u = 10 m/s, T = 318 K
3. Out flow boundary conditions, unknown before calculation
4. The surface of the fin and the outer wall of the tube are calculated by boundary calculation, and the influence of thermal conductivity and surface convection heat transfer is taken into account on these walls
5. The symmetric boundary conditions are adopted because the model is symmetrical in both vertical and horizontal directions
6. Regardless of the influence of the thickness of the tube on the heat conduction, it is considered that the temperature of the outer wall of the tube is the same as that of the inner wall
7. Thickness of each heat exchange tube δ_1_ = 2 mm. Diameter of each heat exchange tube D = 50 mm; Thickness of each fin δ_2_ = 1 mm, the material of finned tube heat exchanger is copper alloy
8. The effects of radiation and thermal resistance are ignored.

The simulated system boundary conditions are set as Table 1^13^:

**Table1:** Boundary conditions of cold source.

Name	Numerical Value/K	Description
4 °C cold source boundary conditions		
T_in	318	Incoming airflow temperature
T_pipe	277	Tube temperature
T_fin	277	Fin temperature
10 °C cold source boundary conditions		
T_in	318	Incoming airflow temperature
T_pipe	283	Tube temperature
T_fin	283	Fin temperature

According to the above conditions, we can complete the steady-state simulation of the finned tube heat exchanger.

### Numerical simulation results and analysis

**Figure 7:**
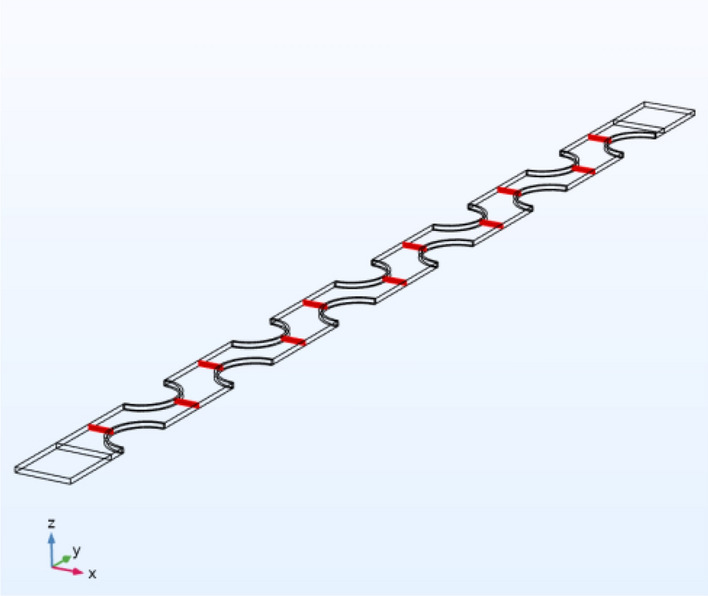
Cross-sectional model of the air field between the fins.

In order to express the simulation results more intuitively. Firstly, 12 sections were added to the internal space of the model, each at a perpendicular angle to the direction of the passing wind flow, and the average temperature of each section was calculated by the simulation software so that the temperature results could be obtained most readily, as shown in Fig. 7.

**Figure 8:**
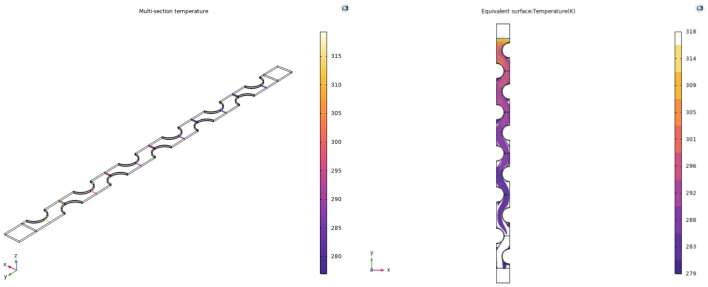
Temperature and equivalent surface temperature of 4 °Ccold source.

The average temperature of the air field cross-section between the fins and the equivalent surface temperature of the 4 °C cooling source are shown in Fig. 8.

**Figure 9:**
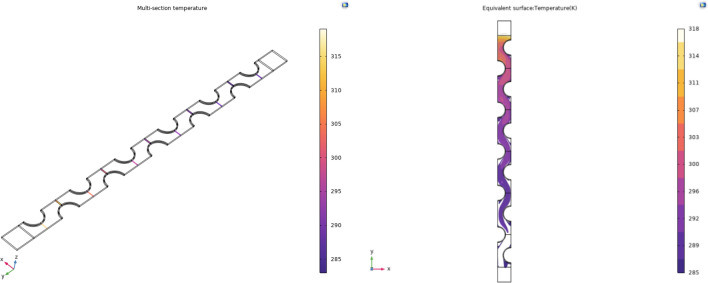
Temperature and equivalent surface temperature of 10 °C cold source.

The average temperature of the air field cross-section between the fins and the equivalent surface temperature of the 10 °C cooling source are shown in Fig. 9.

**Figure 10:**
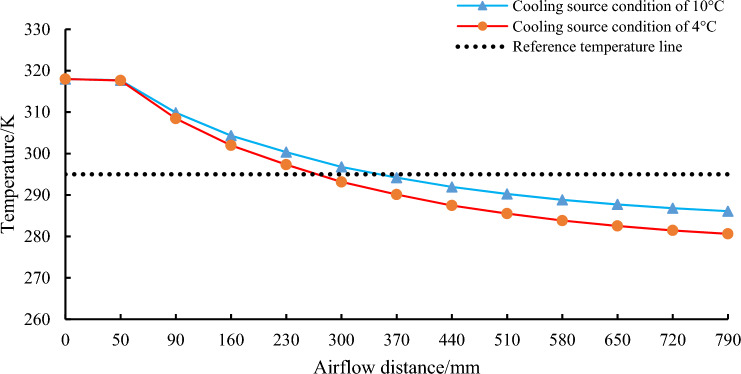
Comparison of the cooling effect of two conditions.

After referring to the actual temperature data of the underground thermal environment of many deep mines, in order to facilitate the design and research needs, we set the cooling wind temperature of the final output of the mobile ice medium cooling equipment at about 295 K, and now use 295 K baseline as the output wind temperature standard. A comparison of the cooling effect of mobile ice media cooling equipment at 4 °C and 10 °C conditions for the cooling source is shown in Fig. 10.

As can be seen from the diagram, if the 4 °C cooling source is used, a heat transfer cooling distance of approximate 260 mm is required to cool the inlet high temperature airflow to the 295 K reference air temperature. If the 10 °C cooling source is used, a heat transfer distance of approximate 310 mm is required to cool down the inlet high temperature airflow to a reference air temperature of 295 K. According to the numerical range of the air field heat transfer and cooling distance between the fins, we determine the minimum number of tube rows required to reach the cooling benchmark temperature, and provide a basis for the design of the fin tube rows of mobile ice medium cooling equipment.

According to Newton's law of cooling and Fourier's law of heat conduction, the heat transfer process of heat conduction and heat convection is directional, and can only be transmitted from a body with a high temperature to a body with a low temperature, or from the hot part of the body to the cold part. It can be seen from the simulation curves of the two types of cold sources that the surface temperature of the tube and fin heat exchanger is much lower than that of the air flow in the roadway. Therefore, according to the second law of thermodynamics, the high temperature air flow through the air field between the fins transfers heat to the cold source in the heat exchange equipment through the heat transfer effect, so the temperature of the air flow is lowered. Mobile ice cooling equipment uses ice as the refrigerant, the specific heat capacity of water is 4.2 J/ (g °C), and the melting heat of ice is 334 J/g. The heat of melting of ice is about 80 times the specific heat capacity of water, this means that the heat absorbed by melting 1 g of ice is equivalent to that required to raise the temperature of 81 g of water by 1 °C under the same conditions. Therefore, the cooling efficiency of the cooling equipment using ice as refrigerant is higher than that of the traditional cold water cooling equipment.

### Application of equipment numerical simulation results

**Figure 11:**
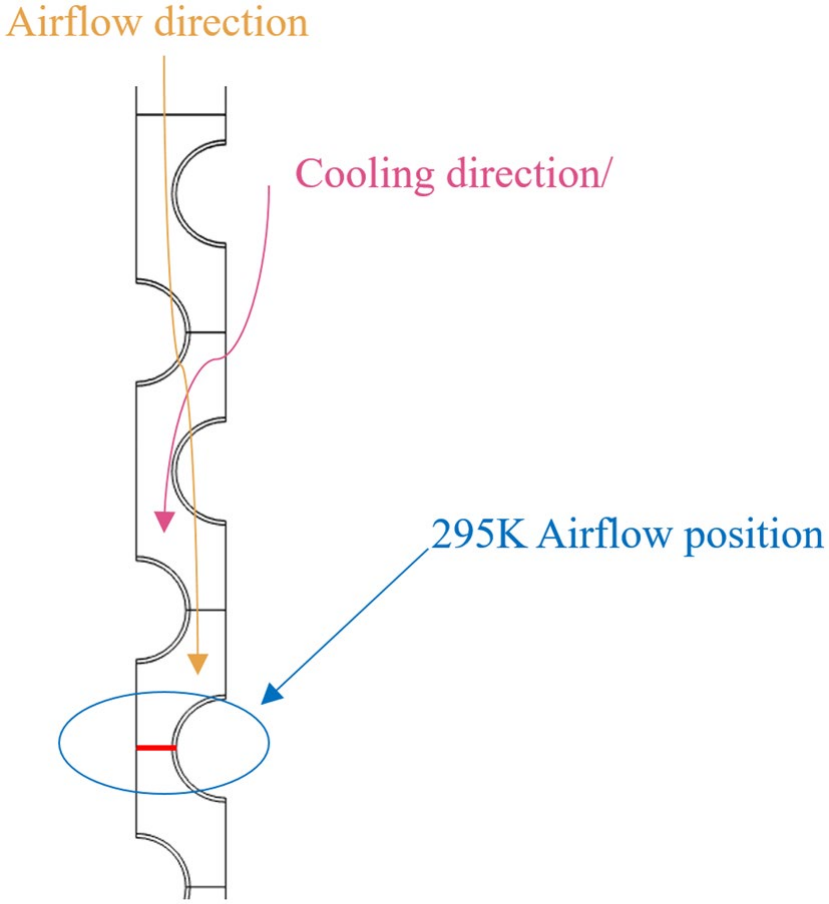
Local area simulation results.

According to the obtained heat transfer and cooling distance, we marked in the numerical model, as shown in Fig. 11, when the red line region is located, we can get the cooling wind with the expected temperature, that is, the wind flow with the temperature of 295 K.

Considering the simulation results, it is imperative to ensure a heat transfer distance of at least 310 mm for the high temperature air flow passing through the inter-fin air field, thereby satisfying the critical cooling distance requirement. Additionally, other influential factors in the heat transfer process should be duly considered. To accomplish this objective, employing a finned tube heat exchanger with 4–10 °C cooling water in the tube, copper alloy material and each heat exchange tube having a diameter (D) of 50 mm would necessitate incorporating no less than five rows of heat exchange tubes.

**Figure 12:**
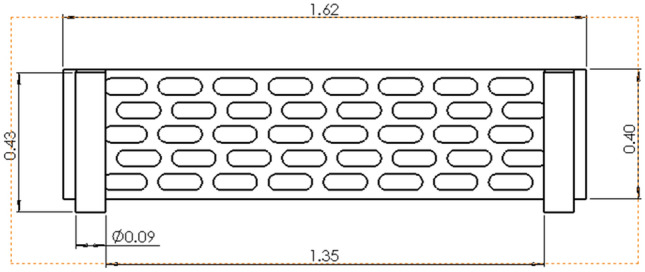
End view of finned tube.

**Figure 13:**
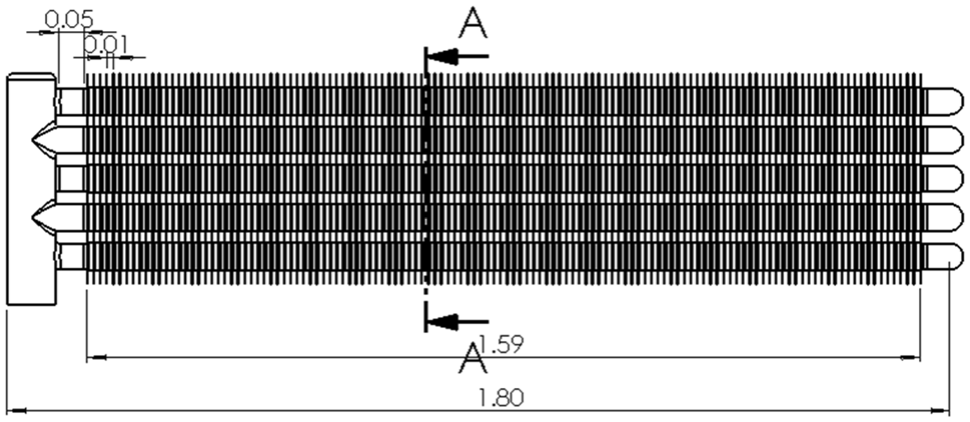
Front view of finned tube.

**Figure 14:**
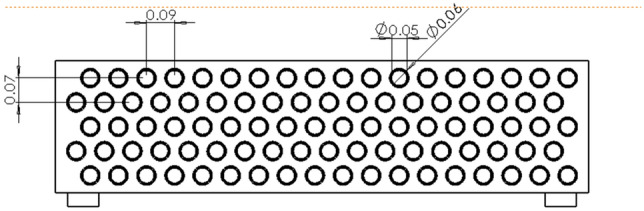
A-A cutaway view of finned tube.

The design layout is shown in Figs. 12, 13 and 14.

## Discussion

The calculation and simulation results demonstrate that the mobile ice medium cooling equipment is capable of delivering a cooling airflow of 295 K to effectively cool the deep roadway in mines. By adhering to the principle of on-demand cooling, this equipment minimizes energy consumption and eliminates unnecessary waste, thereby achieving low operation.

In comparison with large centralized refrigeration systems employed in mines, whether they are ground centralized, underground centralized, or hybrid systems, mobile ice cooling equipment exhibits numerous advantages. Traditional artificial cooling and refrigeration systems encounter challenges such as installation complexities, high investment and maintenance costs, as well as security risks during equipment operation. Conversely, mobile ice cooling equipment utilizes ice as a refrigerant due to its significantly higher latent heat compared to water's specific heat capacity principle enables efficient heat exchange within the system while yielding superior cooling effects.

In addition, the compact size, simplistic structure, flexible layout, and embedded assembly of the equipment offer great convenience. Moreover, it exhibits lower usage and maintenance costs due to its stable and controllable cold source. Additionally, this equipment is minimally affected by external conditions in terms of its operating environment and boasts a wide range of applications. The development of mobile ice media cooling equipment represents a novel exploration in the field of deep ventilation and cooling practices while valuable experiences across various aspects.

The subsequent research of mobile ice cooling equipment will focus on the actual application of engineering experiments by collecting data on the ventilation cooling of the equipment under various underground conditions, and using these data as the basis for studying the cooling law and the actual application effect of the equipment in deep mine, including the best Placement and effective cooling distance to prepare data storage and experiments for subsequent equipment optimization and upgrade. According to the actual requirements of the shaft, simulation is used to study the accurate cooling control of the mobile ice media refrigeration equipment, such as analyzing the cooling effect pattern of the equipment at different temperatures in hot and humid environments, so as to further improve the conversion and usage efficiency of the cold source.

## Conclusions

In an international context emphasizing low carbon and environmental preservation, the provision of on-demand cooling for deep mining holds equal significance. By adopting the principle of on-demand cooling, localized ventilation is employed to cool areas within the underground basic ventilation system, thereby reducing energy consumption. From various research and development endeavors concerning mobile ice media cooling equipment, the following conclusions can be drawn.

The mobile ice cooling equipment exhibits compact size, a simplistic structure, flexible layout, convenient assembly, and cost the inclusion of intricate mechanical and electronic components, thereby ensuring enhanced safety, reliability, and stable performance. In addition, the high temperature air flow passing through the device can be effectively cooled to the required temperature.

From the numerical simulation results, the cooling results and effective cooling distance can be obtained for two critical cold source temperatures. The air temperature inside the inter-fin air field model gradually decreases from the inlet to the interior, indicating that the fins and inter-fin air field of the mobile ice cooling equipment have effective heat transfer efficiency. 4 °C cold source, the inlet 318 K high temperature air flow cooling to 295 K base air temperature requires approximately 260 mm of Heat transfer cooling distance. With the 10 °C cooling source, a heat transfer distance of approximately 310 mm is required to cool down the inlet 318 K high temperature air flow to the 295 K reference air temperature. Based on the cooling results of the high temperature air flow and the capacity factors, the number of finned tubes for the equipment was determined to be 5 rows. These research results provide a demonstration and exploration for the research and development of mobile ice cooling equipment.

### Supplementary Information

Supplementary Information 1.

Supplementary Information 2.

## Data Availability

All data generated or analysed during this study are included in this published article and its supplementary information files. The datasets used and/or analysed during the current study available from the corresponding author on reasonable request.
