# Supplementary material for: Design and analysis of a new type of mobile ice cooling equipment for deep mine
Source: Sci Rep. 2023 Nov 21;13:20375. doi: 10.1038/s41598-023-47902-2 (PMC10663449; doi:10.1038/s41598-023-47902-2)
Supplement: Supplementary file 1 — Supplementary Information 1. [file 41598_2023_47902_MOESM1_ESM.docx]

Raw Data

1.Environmental parameter：

| Wind speed  m/s | Air density  kg/m3 | Air inlet dry bulb temperature  ℃ | Wet bulb temperature at air inlet  ℃ | Relative humidity  % | Local atmosphere  kpa | Dust concentration | Anti-corrosion and wear requirements | Preset temperature of inlet cold water |
| --- | --- | --- | --- | --- | --- | --- | --- | --- |
| 10 | 1.121 | 45 | 34.8 | 99 | 101.325 | Leave aside | Do not consider the use time | 4℃/10℃ |

2.Heat exchanger final parameter selection

Material selection copper

| Fin tube length | Effective length | Fin tube diameter | Wall thickness  δ1 | Segment pitch  d | Thickness  δ2 | Outer diameter of fin  D2 | Effective slice height  d2 | Heat transfer area of a single root | Number of tubes per row | Total heat transfer area |
| --- | --- | --- | --- | --- | --- | --- | --- | --- | --- | --- |
| 1660mm | 1600mm | 50mm | 2mm | 4mm | 0.35mm | 80mm | 15mm | 3.75m^2^ | 10 | 74.95m^2^ |

| Internal distance | 10° | 4° | baseline |
| --- | --- | --- | --- |
| 0 | 318 | 318 | 295 |
| 50 | 317.7 | 317.65 | - |
| 90 | 309.86 | 308.46 | - |
| 160 | 304.33 | 301.99 | - |
| 230 | 300.36 | 297.33 | - |
| 300 | 296.79 | 293.16 | - |
| 370 | 294.19 | 290.11 | - |
| 440 | 291.94 | 287.48 | - |
| 510 | 290.26 | 285.51 | - |
| 580 | 288.83 | 283.82 | - |
| 650 | 287.73 | 282.54 | - |
| 720 | 286.81 | 281.46 | - |
| 790 | 286.1 | 280.63 | - |

3.Raw data of equipment cooling simulation effect
