# Supplementary figures and images for: Design and analysis of a new type of mobile ice cooling equipment for deep mine
Source: Sci Rep. 2023 Nov 21;13:20375. doi: 10.1038/s41598-023-47902-2 (PMC10663449; doi:10.1038/s41598-023-47902-2)

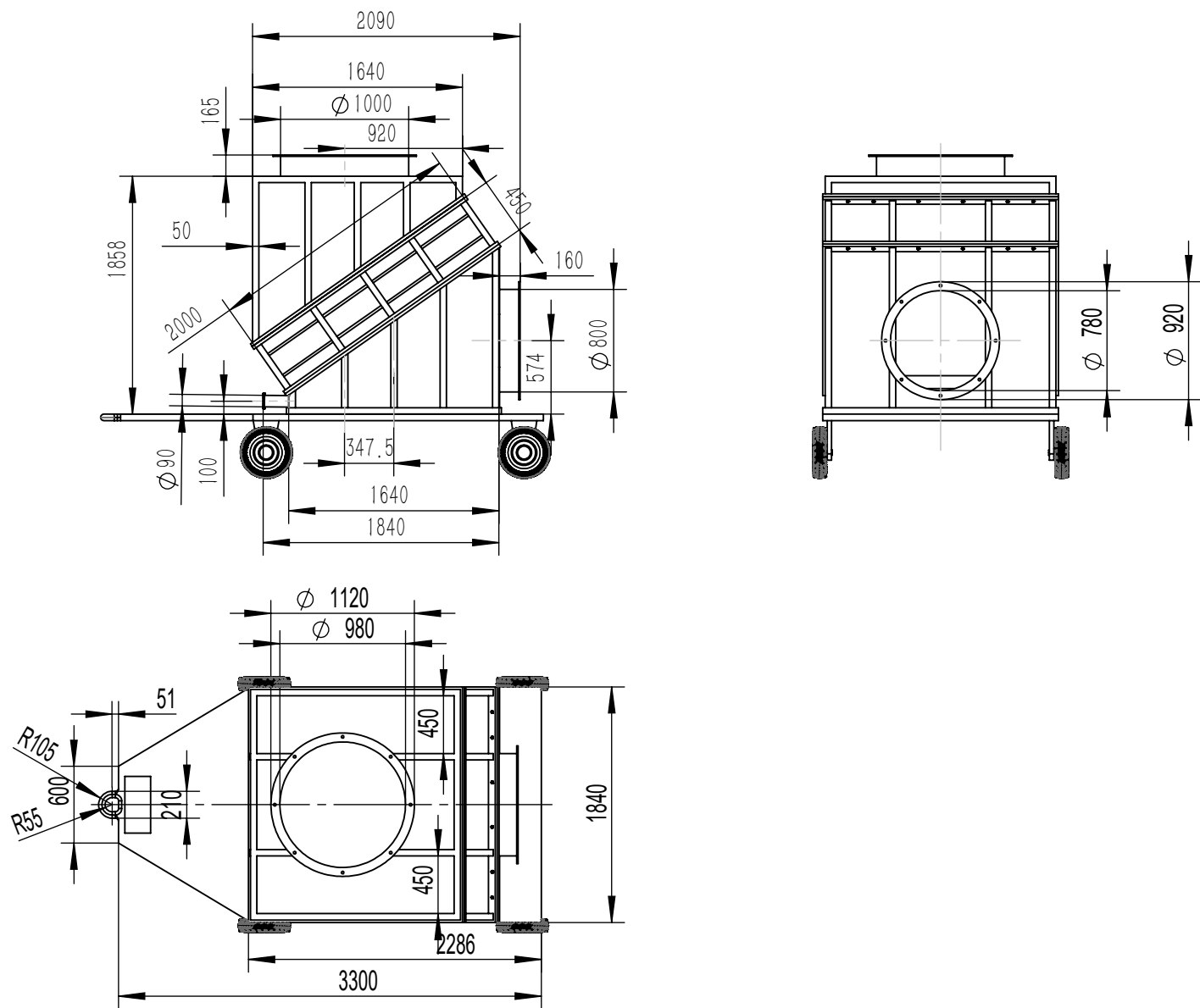

**Main design dimensions of mobile ice cooling equipment**

Supplement: Supplementary file 2 — Supplementary Information 2. [file 41598_2023_47902_MOESM2_ESM.pdf]
